# Cathepsin K associates with lymph node metastasis and poor prognosis in oral squamous cell carcinoma

**DOI:** 10.1186/s12885-018-4315-8

**Published:** 2018-04-05

**Authors:** Frank K. Leusink, Eleftherios Koudounarakis, Michael H. Frank, Ronald Koole, Paul J. van Diest, Stefan M. Willems

**Affiliations:** 1 Department of Head and Neck Oncology and Surgery, Netherlands Cancer Institute – Antoni van Leeuwenhoek, Plesmanlaan 121, 1066 CX Amsterdam, The Netherlands; 2 Department of Oral and Maxillofacial Surgery, University Medical Centre Utrecht, Heidelberglaan 100, 3584 CX Utrecht, The Netherlands; 3 Department of Pathology, University Medical Centre Utrecht, Heidelberglaan 100, 3584 CX Utrecht, The Netherlands

**Keywords:** Oral squamous cell carcinoma, Cathepsin K, Lymph node metastasis, Prognosis

## Abstract

**Background:**

Lymph node metastasis (LNM) is a major determinant of prognosis and treatment planning of oral squamous cell carcinoma (OSCC). Cysteine cathepsins constitute a family of proteolytic enzymes with known role in the degradation of the extracellular matrix. Involvement in pathological processes, such as inflammation and cancer progression, has been proved. The aim of the study was to discover the clinicopathological and prognostic implications of cathepsin K (CTSK) expression in oral squamous cell carcinoma.

**Methods:**

Eighty-three patients with primary OSCC, treated surgically between 1996 and 2000, were included. Gene expression data were acquired from a previously reported study. Human papilloma virus (HPV) status was previously determined by an algorithm for HPV-16. CTSK Protein expression was semi-quantitatively determined by immunohistochemistry in tumor and stromal cells. Expression data were correlated with various clinicopathological variables.

**Results:**

Elevated gene and protein expression of CTSK were strongly associated to LNM and perineural invasion (*p* < 0.01). Logistic regression analysis highlighted increased CTSK protein expression in tumor cells as the most significant independent factor of lymphatic metastasis (OR = 7.65, CI:2.31–23.31, *p* = 0.001). Survival analysis demonstrated CTSK protein expression in both stromal and tumor cells as significant indicators of poor 5-year disease specific survival (HR = 2.40, CI:1.05–5.50, *p* = 0.038 for stromal cells; HR = 2.79, CI:1.02–7.64, *p* = 0.045 for tumor cells).

**Conclusion:**

Upregulation of CTSK seems to be associated with high incidence of lymphatic spread and poor survival in OSCC. CTSK could therefore serve as a predictive biomarker for OSCC.

**Electronic supplementary material:**

The online version of this article (10.1186/s12885-018-4315-8) contains supplementary material, which is available to authorized users.

## Background

Oral squamous cell carcinoma (OSCC) constitutes the most common malignancy of the head and neck region [1]. Lymph node metastasis (LNM) has been shown to be the most significant, independent prognostic factor and is related to a decrease of the 5-year survival rate by 50% [2]. Thus, revealing the presence of occult metastasis is of the utmost importance for early and proper management of the neck. Variable imaging studies have been used for this purpose, including ultrasound combined with fine needle aspiration cytology, computed tomography, magnetic resonance imaging and, more recently, positron emission tomography, with variable results [3]. Moreover, the sentinel node procedure has been currently adopted by some oncological centers and embodied in the staging algorithm of early OSCC. However, its greater disadvantage is that the patient undergoes an interventional procedure. In the context of molecular biology, a significant amount of research has been focused during the last decades on biomarkers that may have additional diagnostic value. Roepman et al. showed that gene expression profiling revealed a strong signature predicting LNM in OSCC [4]. Reanalysis and multicenter validation (*n* = 222) of the entire data set identified more genes with predictive power [5, 6]. Cathepsin K (CTSK) was one of the significantly upregulated genes.

Cathepsin K (also known as cathepsin O2), encoded by the CTSK gene on chromosome 1q21, is one of the 11 lysosomal protein degradation enzymes called cysteine cathepsins, which participate in a considerable number of physiological processes, including MHC-II-mediated antigen presentation, bone remodeling, keratinocyte differentiation and prohormone activation [7]. It is a unique collagenolytic cysteine peptidase and it is highly expressed in osteoclasts and in many other cell types, i.e. macrophages, dendritic cells, adipocytes, fibroblasts and most epithelial cells [8, 9]. Cathepsin K is the sole matrix-degrading enzyme for which a fundamental role in bone resorption has been unequivocally documented in mice and humans [7]. However, increased expression of this lysosomal enzyme is also observed in various pathological conditions, such as neurological disorders, inflammatory diseases and cancer. The role of cathepsins in cancer progression and invasion, mainly through degradation of and remodeling in the tumor microenvironment, is supported by several experimental studies and clinical reports in various types of tumors [10]. In OSCC, both cathepsins B (CTSB) and cathepsin D (CTSD) are correlated with invasion and progression [11, 12] and more specifically CTSD with LNM [12]. Furthermore, CTSB was reported as the promotor of motility and invasiveness [13]. More recently, CTSB was found correlated with survival and LNM, with stronger correlations for the subsite buccal mucosa [14]. Regarding CTSK in OSCC, silencing of CTSK was reported to reduce invasion of aggressive tongue HSC-3 cells in 3D models [15] which could be caused by decreased cell migration and adhesion [16]. To date, there is little data about the relation of CTSK expression in OSCC with clinical and pathological parameters. In the present study, gene and protein expression data of CTKS in OSCC was acquired and the correlation with clinicopathological variables, particularly LNM, was examined.

## Methods

### Patients and tissue samples

**Fig. 1:**
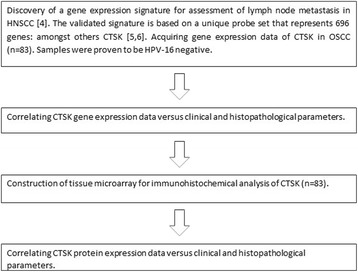
Schematic representation of the work flow of this study. Previous studies resulted in the discovery and validation of a multi-gene signature. In this study, gene expression data were used to correlate the selected gene CTSK with clinical and histopathological parameters. From the same cohort of tumor samples, a TMA was constructed for immunohistochemical analysis of the selected gene to correlate their protein expression with clinical and histopathological parameters, and outcomes were compared

The study work-flow is presented in Fig. 1. The study included 83 consecutive patients with OSCC who were diagnosed and surgically treated at the University Medical Center in Utrecht, The expressioNetherlands, between 1996 and 2000, described in an earlier reported study [17]. Detailed patient characteristics are shown in Table 1. Tissues were used in line with the code ‘Proper Secondary Use of Human Tissue’ as installed by the Dutch Federation of Biomedical Scientific Societies. Table 2 demonstrates the pathological features of the study population. All oral carcinomas included in the current study tested negative for HPV-16 [17], which is in line with other data [18] reporting the incidence of HPV in OSCC to be less than 4%. A previously constructed [17] tissue microarray (TMA) was used for immunohistochemical analysis of CTSK.

**Table 1:** Clinical characteristics of the included OSCC patients

	No.	(%)
All cases	83	(100)
Gender		
Female	36	(43)
Male	47	(57)
Age at diagnosis		
0–60	53	(44)
≥ 61	30	(56)
Median (range)	62	(37–87)
Smoking history		
Current smoker or ceased < 1 year	58	(70)
Ex-smoker, ceased > 1 year	9	(11)
Never smoker	15	(18)
Alcohol consumption		
≥ 5 U/day	19	(23)
1–4 U/day	28	(34)
Occasionally	17	(20)
Never	19	(23)
Clinical T-stage		
cT1	13	(16)
cT2	31	(37)
cT3	8	(10)
cT4	31	(37)
Clinical N-Stage		
cN0	53	(64)
cN1–3	30	(36)
Sub-site		
Tongue	30	(36)
Floor of mouth	35	(42)
Buccal cavity	10	(12)
Gum	8	(10)
Mean follow-up (months)	45

**Table 2:** Pathological characteristics of the included OSCC patients

	No.	(%)
All cases	83	(100)
Pathological T-Stage		
pT1	17	(20)
pT2	27	(33)
pT3	10	(12)
pT4	29	(35)
Pathological N-Stage		
pN0	38	(46)
pN1–3	45	(54)
Stage grouping		
I	14	(17)
II	9	(11)
III	22	(26)
IVA-IVB	38	(46)
Infiltration depth		
≥ 4.0 mm	72	(87)
< 4.0 mm	11	(13)
Differentiation grade		
Good / Moderate	67	(81)
Poor / Undifferentiated	16	(19)
Keratinization		
Present	60	(72)
Absent	20	(24)
Missing	3	(4)
Vaso-invasion		
Present	18	(22)
Absent	62	(75)
Missing	3	(3)
Bone-invasion		
Present	25	(30)
Absent	58	(70)
Perineural growth		
Present	34	(41)
Absent	39	(47)
Missing	10	(12)
Spidery growth		
Present	65	(78)
Absent	18	(22)
High risk HPV status		
positive	0	(0)
negative	83	(100)

### CTSK gene expression analysis

Genome-wide gene expression was measured using dual-channel microarrays with a pool of tumor samples, as described in an earlier study [4]. CTSK was represented by a sole, unique feature on this array.

### Immunohistochemistry

Five μm thick sections of FFPE tonsil control tissue and the TMA were cut and mounted on silane-coated glass slides. After deparaffinization the endogenous peroxidase activity was blocked for 30 min in a 0.3% H_2_O_2_ phosphate-citrate buffer solution of pH 5.8 with sodium azide. Then, tissue sections were subjected to antigen retrieval by boiling in sodium citrate solution (pH 6) for 15 min at 37 °C. Subsequently, the tissue slides were washed with PBS, 0.05% (*v*/v) Tween-20 and incubated with a dilution of the primary mouse monoclonal antibody against CTSK (clone CK4, Novocastra, Newcastle, UK) for 1 h at RT. Slides were washed and incubated with the following species-specific secondary antibodies: 1:250 diluted rabbit anti-mouse (RAMPO, Dako, Glostrup, Denmark) followed by Powervision anti-rabbit/HRP conjugated (Klinipath, Duiven, The Netherlands). All antibodies were diluted in PBS/1%BSA. After washing with PBS, the bound antibodies were visualized using 3,3′-diaminobenzidine (0.6 mg/ml). Slides were counterstained with hematoxylin.

### Evaluation of protein expression

**Fig. 2:**
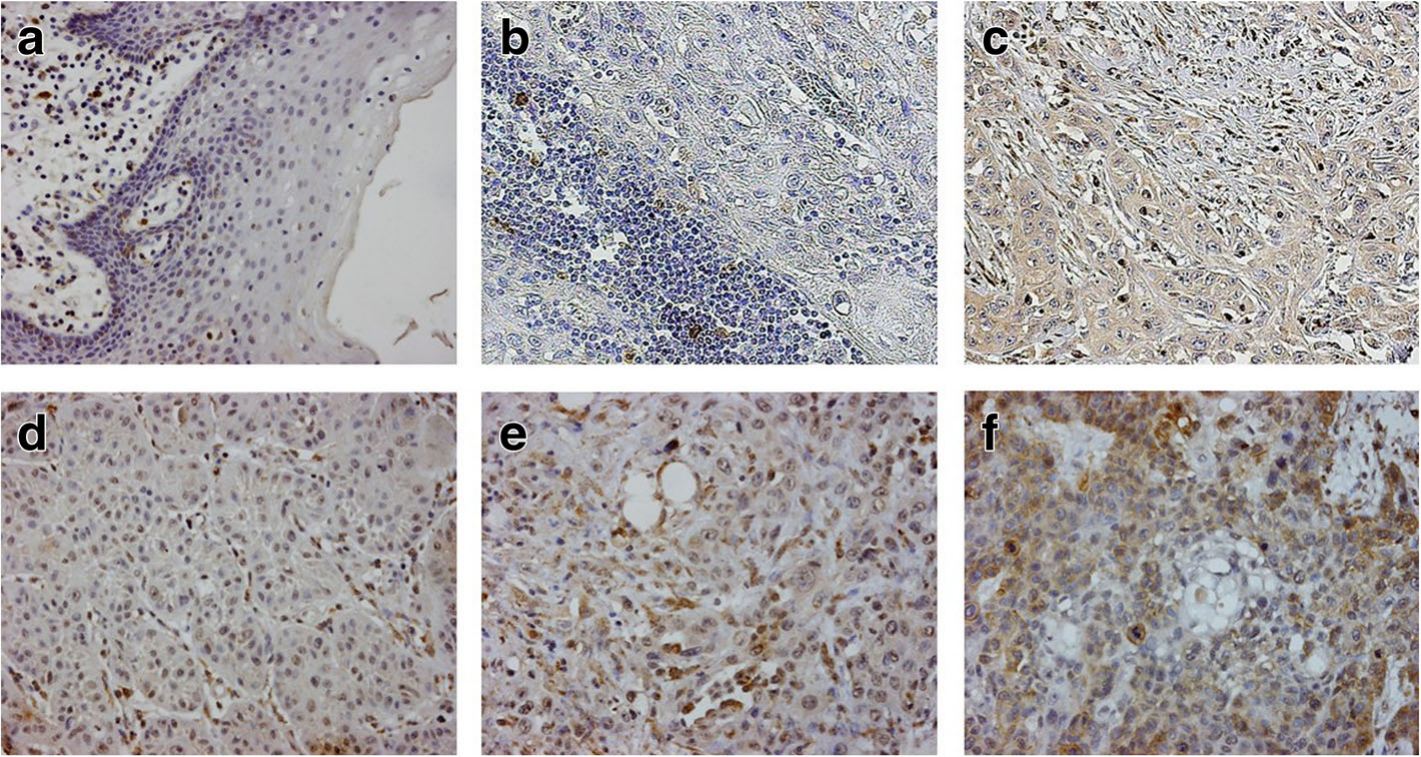
CTSK expression in OSCC and normal mucosa. Representative stainings of the TMA, consisting of 83 OSCC cases, are presented. CTSK is diffusely expressed, is stained both in tumor as in stromal cells and varies in expression from non to strong expression. Staining scores were calculated by the product of intensity (normal = 1, strong = 2) and the proportion of stained tumor or stromal cells (%). Panels **a**-**f** represent examples of CTSK staining; **a**) normal mucosa, **b**) OSCC negative for CTSK in stromal cells, **c**) OSCC with normal staining in tumor cells and in stromal cells, **d**) OSCC negative for CTSK, **e**) OSCC with a normal intensity (score = 1 × 50% = 50), **f**) OSCC with a strong intensity (score = 2 × 75% = 150)

Intensity and percentages of positive tumor cells were semi-quantitatively and independently evaluated by 3 observers (SMW, PJvD and FKL) who were blinded to patient outcome. Stromal positive cells were evaluated separately in a likewise fashion. In case of disagreement, the observers reanalyzed the staining results until they reached consensus. To determine the score for each TMA-core, appropriate controls of normal squamous epithelium were used. Protein expression was scored for both its intensity in tumor cells relative to normal epithelium (strong expression = 2, normal expression = 1, no expression = 0) and the percentage of tumor cells in the tissue section with such a specific intensity. Multiplying of these two scoring variables resulted in a scoring range of 0 up to 200, in which a score of 0 represents a complete loss or no expression of protein in all tumor cells and a score of 200 represents a high expression throughout the tumor (Fig. 2a-d). Cores were considered lost if less than 10% of cells contained tumor (‘sampling error’) or when less than 10% of tissue was present (‘absent core’). Cases were excluded if more than 2 cores were lost per case. When the scores between the cores of a particular case differed, the most frequent score determined the overall score. In case of 4 different scores in one case, the average score was calculated.

### Statistical analysis

The non-parametric Mann-Whitney U test was used to determine differences in CTSK expression between various clinicopathologically defined groups. Logistic regression techniques were used to assess correlations between CTSK expression and the incidence of neck LNM. Overall survival (OS) was defined as the length of the time from surgery to death from any cause. Disease-specific survival (DSS) was defined as the time from surgery to death due to disease. Receiver operating characteristic (ROC) curves were designed to determine optimal cut-off values. The association between CTSK and the primary and secondary outcomes was analysed with crosstabs, chi-square test (or Fisher’s Exact Test when appropriate), logistic regression, Kaplan Meier/logrank survival analyses, and Cox-regression.

All *p* values were based on two-tailed statistical analysis and *p* < 0.05 was considered significant. Statistical analysis was performed using the SPSS 25.0 statistical package (IBM Corp. IBM SPSS Statistics for OSx, Version 25.0. Armonk, NY: IBM Corp).

## Results

### Gene expression and clinicopathological variables

A statistically significant association of high CTSK mRNA levels to lymphatic metastasis (*p* < 0.01) was observed, as wells as to vaso- and perineural invasion (*p* < 0.01 in both cases; Table 3). In contrast, no significant correlation was found to other pathological characteristics, such as pT status, depth of invasion and tumor grade.

**Table 3:** Correlations between gene (mRNA) and protein (IHC) expression of CTSK and clinical and pathological parameters of the included OSCC cohort (*n* = 83)

		CTSK	
	mRNA	IHC tumor	IHC stroma
Clinical characteristic			
Smoking history	NS	NS	NS
Alcohol consumption	*p* < 0.01	NS	NS
Age	NS	NS	NS
cT status	NS	NS	NS
cN status	NS	NS	NS
Subsite	NS	NS	NS
Pathological characteristic			
pN-status	*p* < 0.01	*p* < 0.01	*p* < 0.01
pT-status	NS	NS	NS
Infiltration depth	NS	NS	NS
Differentiation grade	NS	NS	NS
Vaso-invasion	*p* < 0.01	NS	NS
Bone-invasion	NS	NS	NS
Peri-neural invasion	*p* < 0.01	*p* < 0.01	NS
Spidery growth	NS	NS	NS

Cases were stratified according to clinical and pathological characteristics. Smoking history was dichotomized to current smoker or ceased < 1 year versus ex-smoker (ceased > 1 year) and never smoker. Alcohol consumption was dichotomized to 1–4 or ≥ 5 U/day versus occasionally or never. Clinical and pathological nodal status (cN and pN) were dichotomized to cN0 versus cN+ and to pN0 versus pN+. Infiltration was dichotomized to < 4 mm versus ≥4 mm. Differentiation was dichotomized to well and moderate versus poor and undifferentiated. *P*-values represent the Mann-Whitney U test of these comparisons. IHC: immunohistochemistry; mRNA: messenger ribonucleic acid; NS: non-significant

Among the various clinical parameters, a strong correlation of increased gene expression was found only to alcohol consumption (*p* < 0.01). No significant relationship was found to smoking history, age, tumor subsite and clinical T or N stage.

### CTSK gene expression and survival

**Fig. 3:**
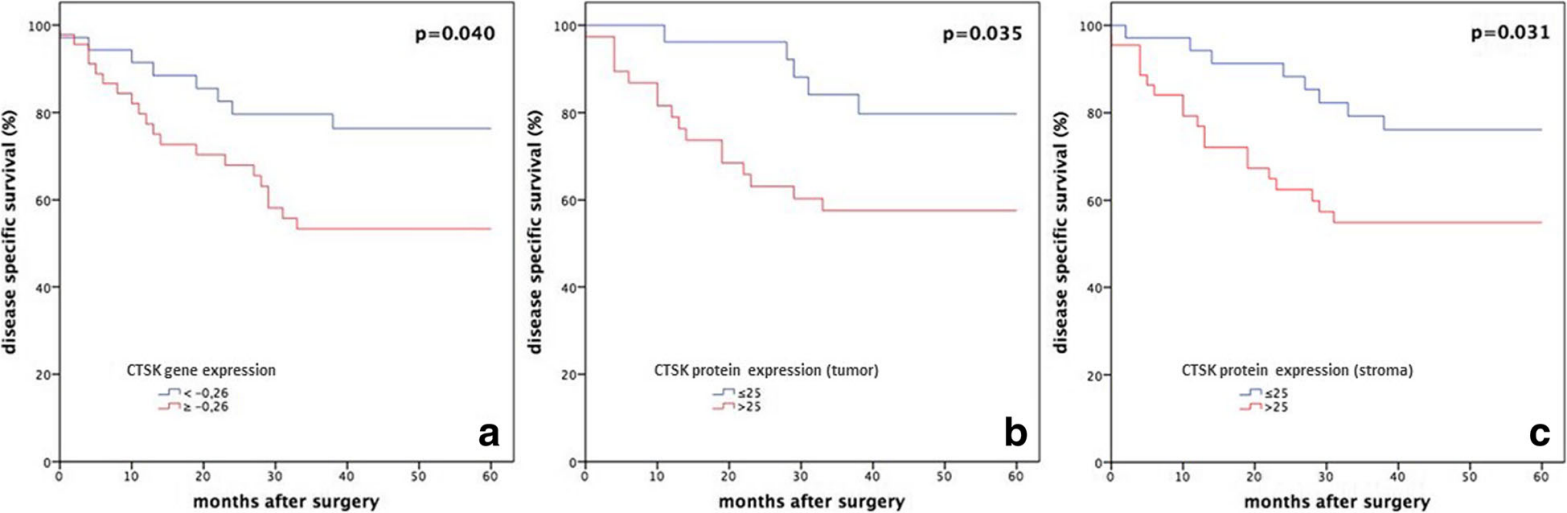
Kaplan Meier disease specific survival (DSS) plots for all patients with OSCC (*n* = 83). Cases were stratified according to differential expression of CTSK, and were dichotomized into low and high expression according to the determined cut-off point in panel **a** for gene expression (− 0.26) and in panel **b** and **c** for protein expression (25). *P*-values in **a**-**c** represent the Log-rank test of this group comparison and therefore differ from the significance levels of the Cox-regression analysis in Table 4. In all three analyses, high CTSK expression was strongly associated with a worse 5-year DSS

A Cox regression analysis was performed in order to determine the prognostic significance of the CTSK gene expression. Dichotomization was based on the cut-off value of − 0.26, determined by ROC analysis. Patients with high CTSK gene expression demonstrated a significantly worse 5-year DSS (HR = 2.29, CI:1.01–5.21, *p* = 0.047; Table 4). The pathological N status was shown to have the strongest impact for DSS (HR = 4.10, CI:1.66–10.15, *p* = 0.002). The prognostic significance of CTSK gene expression did not hold for overall survival (OS) (*p* = 0.2). The Kaplan-Meier survival plot is shown in Fig. 3a (*p* = 0.040).

**Table 4:** Univariate and multivariate DSS Cox regression model for gene and protein CTSK expression

	Univariate		
	HR	95% CI	*p*-value
Age^a^	1.01	0.48–2.12	0.978
Tumor stage^b^	4.01	1.21–13.29	0.023
pN^c^	4.10	1.66–10.15	0.002
CTSK protein expression (stroma)	2.40	1.05–5.50	0.038
CSTK protein expression (tumor)	2.79	1.02–7.64	0.045
CTSK gene expression	2.29	1.01–5.21	0.047
	Multivariate		
pN status	3.61	1.12–11.57	0.030
corrected for CTSK protein expression (tumor)			

Dichotomization was made according to the cut-off values into high and low expression. The most important prognostic parameters (age, stage and pN) were added in the regression model

^a^< 60 vs. ≥60 years; ^b^I, II vs. III, IV; c pN0 vs. pN+

### CTSK protein expression and clinicopathological variables

A total of 213 (64%) cores with tumor and 246 (74%) cores with stroma stained with the CTSK antibody were available for analysis. Due to our inclusion criteria (≥2 tumor cores available per case), 19 cases were missing. The majority of the OSCCs in this cohort showed a weak expression for CTSK (42% in tumor cells and 54% in stromal cells), whereas only 5% demonstrated no expression in tumor cells. All stromal samples showed some expression (Additional file 1: Table S1).

A cut-off value of 25 was determined by ROC analysis, in order to divide patients into low and high protein expression groups. No statistically significant correlation to clinical variables was found (Table 3). In contrast, there was a significant association with histopathologically proven LNM (*p* < 0.01) and increased CTSK expression in both tumor and stromal cells. A similar strong relationship to peri-neural invasion was also demonstrated (*p* = 0.01) for CTSK tumor cell expression. No association to other pathological variables was evident.

In logistic regression analysis, factors with known impact to nodal disease were incorporated into the model, including T stage, perineural and vaso-invasion, depth of infiltration and spidery growth pattern, along with CTSK protein expression in tumor and in stroma cells (Table 5). In univariate analysis, high CTSK expression (tumor and stroma) appeared to be an important independent predictive factor of lymph node involvement (tumor: OR = 7.65, CI: 2.51–23.32; *p* < 0.001 and stroma: OR = 4.04, CI: 1.57–10.36; *p* = 0.004). In multivariate analysis, CTSK protein expression (tumor), corrected for pathological T stage, remained a strong prognostic factor for regional disease, demonstrating an odds ratio of 9 (CI: 2.83–31.65; *p* < 0.01).

**Table 5:** Univariate and multivariate logistic regression model for CTSK protein expression and LNM

	Univariate		
	OR	95% CI	*p*-value
pT^a^	1.49	1.01 - 2.20	0.044
Peri-neural invasion^a^	5.20	1.87–14.45	0.002
Vaso-invasion^a^	9.71	2.06–45.89	0.004
Depth of invasion	1.51	0.85–2.69	0.165
Spidery growth	4.44	1.41–14.00	0.011
CTSK protein expression (stroma)	4.04	1.57–10.36	0.004
CTSK protein expression (tumor)	7.65	2.51–23.32	< 0.001
	Multivariate		
CTSK protein expression (tumor)	9.46	2.83–31.65	< 0.01
corrected for pT-status			

Dichotomization was made into low expression (score 0–25) versus high expression (score 26–200). The most important predictive parameters (pT, peri-neural invasion, vaso-invasion, depth of invasion, spidery growth) were added in the model

LNM = lymph node metastasis, OR = odds ratio, HR = hazard ratio, 95% CI = 95% confidence interval, *p*-value of the Cox regression model

^a^age: < 60 vs ≥ 60 years, tumor stage I, II vs III, IV, pN status pN0 vs pN+

Next, the predictive value of CTSK as a biomarker of occult metastasis in early stage (cT1-T2 N0) OSCC was examined. A total of 24 patients had early T stage without clinically detectable nodal disease. Out of the ten patients with yet occult metastases in the neck dissection specimen, nine had a high protein CTSK expression, whereas only one patient showed a value lower than the cut-off (Table 6). The sensitivity of high protein expression in detecting occult metastasis in early stage OSCC was, thus, calculated at 90%, whereas the specificity was 57%. Additionally, the positive predictive value was found at 60%, with a negative predictive value of 89%.

**Table 6:** Allocation of cT1-T2 N0 patients based on their pathological N-status and CTSK protein expression

		pN status		Total
		pN0	pN+	
CTSK	≤25 > 25	8 6	1 9	9 15
Total		14	10	24

The value of CTSK protein expression in predicting occult metastasis (≤25 predicts pN0, > 25 predicts pN+) in cT1-T2 N0 patients is calculated as follows: sensitivity (9/9 + 1) × 100% = 90%; specificity (8 + 6) × 100% =57%; positive predictive value (9/6 + 9) × 100% = 60%; negative predictive value (8/8 + 1) × 100% = 89%

### CTSK protein expression and survival

In Cox regression, CTSK protein expression of tumor and stromal cells was dichotomized into low versus high based on the previously reported cut-off value, demonstrating a significantly worse DSS in OSCC subjects with increased CTSK protein expression (tumor: HR = 2.79, CI 1.02–7.64, *p* = 0.045 and stroma: HR = 2.40, CI 1.05–5.50, *p* = 0.038; Table 4). No prognostic impact on overall survival was found. The Kaplan-Meier survival plot is shown in Fig. 3b (*p* = 0.035, tumor) and in Fig. 3c (*p* = 0.031, stroma).

In multivariate analysis, pN status was corrected for CTSK protein expression (tumor), and pathological N status showed once more a strong correlation (HR = 3.61, CI:1.12–11.57, p = 0.03), with a change though of the beta coefficient greater than 10%, confirming the role of CTSK as a significant confounder for DSS.

## Discussion

Cancer metastasis is a complex process that includes a number of different events, referred as the invasion-metastasis cascade. The first critical step of the process is the invasion of the malignant cells into the surrounding extracellular matrix (ECM) and stromal cell layers [19]**.** The biological role of CTSK in promoting tumor invasion and migration has been proved ex vivo in cell-based systems [15, 16]**.** Apart from their well-known function of ECM degradation and remodeling, cathepsins are also suggested to participate in the activation cascade of pro-urokinase-type plasminogen activator and other proteases, enhancing thus their effect in the dissolution of the tumor matrix and basic membrane [20]. In addition to their extracellular function, there is evidence that intracellular cathepsins promote tumor progression by affecting processes acting both as pro-tumorigenic and anti-tumorigenic [21]. Intracellular collagen degradation is an example of the potential intracellular pro-tumorigenic activity of cathepsins.

The pathophysiological role of the cathepsins in cancer metastasis has attracted the interest of studying its value as a biomarker of metastasis and prognosis in various types of malignancy. Increased protein expression of cathepsins V, B and D has been associated with distant metastasis and worse DSS in breast cancer [22]. Similar results have been found for cathepsin A in malignant melanoma and cathepsin B in non-small cell lung carcinoma [23, 24] and in OSCC [11–14]. Overexpression of CTSK has been observed in invasive ductal carcinoma of the breast, lung and prostate adenocarcinoma [25–27]. In all these studies, increased protein expression was related to high metastatic potential. Interestingly, high expression of CTSK was found in the desmoplastic reactive stroma of the lung adenocarcinoma, indicating that stromal production of CTSK can favor or modulate the invasion of tumor cells [28]. In oral and oropharyngeal SCC, downregulation of the cognate inhibitor of CTSK [29], SERPINB13, was reported to be associated with LNM and poor prognosis [30]. However, only one study exists in the literature regarding the prognostic value of CTSK in tongue SCC [15]. In that study, Bitu et al. reported that CTSK was expressed in both stromal and tumor cells by immunohistochemistry. The only significant finding was that CTSK expression in stromal cells exhibited a potential protective role, since a poorer prognosis in early stage tumors was correlated to weak CTSK expression in the tumor microenvironment front. However, the same study found decreased invasion of HSC-3 tumor cells when cathepsin K silencing was applied. It was, thus, concluded that different prognosis could be exhibited, depending on whether CTSK is expressed more in tumor or stromal cells.

The present study is the first conducted to explore the predictive and prognostic value of CTSK in patients with OSCC. Combined evaluation of both gene and protein expression was used to augment the validity of clinicopathological correlations. Although some discrepancies were found in the associations with the clinicopathological parameters between gene and protein expression, the results are likewise. Some variation may be expected since mRNA levels are not directly proportional to the protein concentration due to post-translational mechanisms, that control protein turnover and abundance, and different translational rates, which are determined by constants that are not completely known [31]. Another factor could also be that gene expression data were acquired using biopsies taken at the border of the primary tumor and samples were included if they consisted of at least 50% tumor cells. The other part of the sample consisted of stromal cells and epithelial cells adjacent to the tumor. Consequently, gene expression was measured using tumor, stromal and epithelial cells, whereas protein expression was scored semi-quantitatively by immunoreactivity in tumor cells and in stromal cells separately. Finally, the semi-quantitative nature of immunoreactivity could cause a potential difficulty in accurate measurement of CTKS levels in paraffin tissue compared to the mRNA CTSK levels determined in fresh frozen biopsies. This could be another reason for the discrepancy between mRNA and protein data.

There are various explanations for the disagreement with the results reported by Bitu et al. Our findings show that CTSK expressed in either tumor cells or stroma cells correlates with a higher risk for lymph node metastases and a worse DSS. This corresponds with other reports on CTSK expression in different tumor entities [28] but is discrepant with Bitu et al. suggesting that stromal CTSK seems to have a protective role in the complex progression of tongue cancer. After scoring CTSK staining of both tumor cells and of the tumor micro environment (TME), Bitu determined staining gradients (TME: tumor) into higher, lower or no gradient. ‘No gradient’ can only be achieved if there is complete lack of TME staining. Next, they combined in the survival analyses ‘no gradient’ samples with ‘higher gradient’ samples to be compared with the ‘lower gradient’ samples which is biologically inappropriate, but explains their suggestion and the discrepancy. Although not reported, there were probably more ‘no gradient’ samples than ‘higher gradient’ samples combined and compared in the survival analyses with ‘lower gradient’ samples. Furthermore, the different scoring system as well as the different antibody clone used to detect cathepsin K could play a role. In the previous study, there is also insufficient information about the diagnostic approach to the lymph node status and incomplete pathological data, such as infiltration depth and perineural invasion, of the studied cohort. Lack of these data could underestimate the clinicopathological correlations. Third, the findings of the previous study were solely based on immunohistochemistry and were, partly, contradictory with the observation, reported by the same authors, of the diminished invasion potential of the HSC-3 tumor cells, when cathepsin K was silenced or inhibited.

The results of the current study suggest that CTSK may be used as a predictive biomarker in patients with OSCC. Its high sensitivity (90%) combined to the high negative predictive value (89%) makes it particularly valuable in excluding occult metastasis in early T1-2 N0 OSCC, allowing to perhaps rely on a “wait and see” policy for the management of the neck. Moreover, it is shown that tumors with up-regulation of CTSK harbored a high potential for perineural invasion. This can be interpreted by the proteolytic action on the nerves’ epineurium and perineurium, facilitating tumor cell migration into the nerve fasciculus. Hence, CTSK can be a molecular determinant of perineural invasion, apart from the various neurotrophins and chemokines that are involved in this process [32]. The strong relationship of CTSK with both lymphatic spread and perineural invasion is also reflected by its significant impact on DSS.

The current study was based on a relatively limited cohort of 83 patients with OSCC. The results should be further validated by studies using different CTSK antibodies and including higher number of patients with emphasis on predicting occult metastasis in cases of N0 stage. Furthermore, serum levels of CTSK can be also evaluated at different stages of the disease and correlate them to clinicopathological variables. Important prognostic implications of elevated serum levels of cathepsins have been observed in other types of malignancies, such as in prostate cancer [33]. Finally, the emergence of new CTSK inhibitors, like odanacatib [16], can provide in the future a new tool for the suppression of tumor progression in patients with inoperable disease.

## Conclusion

In conclusion, our findings provide evidence that increased CTSK expression is associated with lymphatic spread and poor prognosis of OSCC. Due to the high negative predictive value (89%) of CTSK protein expression, this biomarker can be a simple and useful tool in the diagnostic work-up of cT1-T2 N0 OSCC, however it should be validated first in a larger prospective cohort study.

## Additional file

Additional file 1: **Table S1.** Immunohistochemical descriptive results of protein expression of CTSK in the OSCC TMA cohort (*n* = 83). (DOCX 14 kb)

## Abbreviations

CI: Confidence interval
CTSK: Cathepsin K
DSS: Disease-specific survival
FFPE: Formalin-fixed, paraffin-embedded
HE: Hematoxylin and eosin
HPV: Human papilloma virus
HR: Hazard ratio
IHC: Immunohistochemistry
LNM: Lymph node metastasis
OR: Odds ratio
OS: Overall survival
OSCC: Oral squamous cell carcinoma
PCR: Polymerase chain reaction
ROC: Receiver operating characteristic
TMA: Tissue microarray
