# Supplementary material for: Cathepsin K associates with lymph node metastasis and poor prognosis in oral squamous cell carcinoma
Source: BMC Cancer. 2018 Apr 5;18:385. doi: 10.1186/s12885-018-4315-8 (PMC5885370; doi:10.1186/s12885-018-4315-8)
Supplement: Supplementary file 1 — Table S1. Immunohistochemical descriptive results of protein expression of CTSK in the OSCC TMA cohort (n = 83). (DOCX 14 kb) [file 12885_2018_4315_MOESM1_ESM.docx]

**Additional File 1**

**Table S1.** Immunohistochemical descriptive results of protein expression of CTSK in the OSCC TMA cohort (n=83).

| Variable | | CTSK_tumor | | | | | CTSK_stroma | | |  | |  | |  | |
| --- | --- | --- | --- | --- | --- | --- | --- | --- | --- | --- | --- | --- | --- | --- | --- |
| Number (%) of 332 cores | |  | |  | |  | | |  |  |  |  |  |  |  |
| Tumor / stroma | | 213 | | (64) | | 246 | | | (74) |  |  |  |  |  |  |
| No tumor / stroma | | 68 | | (20) | | 32 | | | (10) |  |  |  |  |  |  |
| No core | | 51 | | (15) | | 54 | | | (16) |  |  |  |  |  |  |
| Number of cores / case (n=83) | |  | |  | |  | | |  |  |  |  |  |  |  |
| 4 | | 23 | | (28) | | 38 | | | (46) |  |  |  |  |  |  |
| 3 | | 29 | | (35) | | 21 | | | (25) |  |  |  |  |  |  |
| 2 | | 12 | | (14) | | 10 | | | (12) |  |  |  |  |  |  |
| 1 | | 11 | | (13) | | 10 | | | (12) |  |  |  |  |  |  |
| 0 | | 8 | | (10) | | 4 | | | (5) |  |  |  |  |  |  |
| Score (%) / case (n=83) | |  | |  | |  | | |  |  |  |  |  |  |  |
| 0 | (no expression) | 4 | | (5) | | 0 | | | (0) |  |  |  |  |  |  |
| 1-50 | (weak expression) | 35 | | (42) | | 45 | | | (54) |  |  |  |  |  |  |
| 51-200 | (strong expression) | 26 | | (31) | | 34 | | | (41) |  |  |  |  |  |  |
| missing |  | 18 | | (22) | | 4 | | | (5) |  |  |  |  |  |  |
|  | |  |  | |  | | |  | |  |  |  |  |  |  |

Percentages may not total 100 because of rounding.
